# Huntington’s disease blood and brain show a common gene expression pattern and share an immune signature with Alzheimer’s disease

**DOI:** 10.1038/srep44849

**Published:** 2017-03-21

**Authors:** Davina J. Hensman Moss, Michael D. Flower, Kitty K. Lo, James R. C. Miller, Gert-Jan B. van Ommen, Peter A. C. ’t Hoen, Timothy C. Stone, Amelia Guinee, Douglas R. Langbehn, Lesley Jones, Vincent Plagnol, Willeke M. C. van Roon-Mom, Peter Holmans, Sarah J. Tabrizi

**Affiliations:** 1 Department of Neurodegenerative Disease, University College London Institute of Neurology, London, WC1B 5EH, UK; 2 University College London Genetics Institute, University College London, London, WC1E 6BT, UK; 3 Department of Human Genetics, Leiden University Medical Center, Leiden, Postzone S-4-P, The Netherlands; 4 MRC Centre for Neuropsychiatric Genetics and Genomics, School of Medicine, Cardiff University, CF24 4HQ, UK; 5 Faculty of Education, University of Cambridge, CB2 8PQ, Cambridge UK; 6 Departments of Psychiatry and Biostatistics, University of Iowa, IA 52242, USA

## Abstract

There is widespread transcriptional dysregulation in Huntington’s disease (HD) brain, but analysis is inevitably limited by advanced disease and postmortem changes. However, mutant *HTT* is ubiquitously expressed and acts systemically, meaning blood, which is readily available and contains cells that are dysfunctional in HD, could act as a surrogate for brain tissue. We conducted an RNA-Seq transcriptomic analysis using whole blood from two HD cohorts, and performed gene set enrichment analysis using public databases and weighted correlation network analysis modules from HD and control brain datasets. We identified dysregulated gene sets in blood that replicated in the independent cohorts, correlated with disease severity, corresponded to the most significantly dysregulated modules in the HD caudate, the most prominently affected brain region, and significantly overlapped with the transcriptional signature of HD myeloid cells. High-throughput sequencing technologies and use of gene sets likely surmounted the limitations of previously inconsistent HD blood expression studies. Our results suggest transcription is disrupted in peripheral cells in HD through mechanisms that parallel those in brain. Immune upregulation in HD overlapped with Alzheimer’s disease, suggesting a common pathogenic mechanism involving macrophage phagocytosis and microglial synaptic pruning, and raises the potential for shared therapeutic approaches.

Huntington’s disease (HD), the most common monogenic neurodegenerative disorder in the developed world^1^, is caused by a CAG repeat expansion in the *HTT* gene and is characterised by motor, cognitive and psychiatric features. Onset occurs around 45 years on average and inversely correlates with CAG repeat length^2^. The disease progresses inexorably and, with the exception of late-onset cases, is uniformly fatal a median of 18 years from motor onset^3^. HD is currently incurable and no treatments slow progression.

HD research has traditionally focused on the brain due to the presence of characteristic mutant huntingtin protein aggregates^4^ and because the prominent symptoms and signs can be linked to neurodegeneration in the basal ganglia and cerebral cortex^5^. However, mutant *HTT* is ubiquitously expressed^6^ and mounting evidence suggests it has direct effects in peripheral tissues^5^,^7^, though whether these effects are distinct, or parallel those in the brain remains unclear. HD patients demonstrate peripheral immune dysfunction presymptomatically^8^,^9^,^10^,^11^, as well as weight loss that leads to cachexia with advancing disease^7^. There is progressive muscle wasting^12^, endocrine dysfunction^13^ liver impairment^7^, and cardiac dysfunction^14^,^15^,^16^. Mutant HTT protein aggregates can be found in the peripheral tissues of HD mice^17^, as well as advanced patients^18^. These peripheral features may contribute to CNS pathology, disease progression and mortality^5^,^7^, and strongly suggest that HD is a systemic disorder. This peripheral phenotype provides an opportunity to study mutant huntingtin’s pathogenic mechanisms. In contrast to brain tissue, availability of which is limited and from post-mortem subjects with end-stage disease^19^,^20^, peripheral tissues can be sampled minimally invasively and inexpensively from living patients, enabling longitudinal study throughout disease course.

Transcriptional dysregulation is a central feature of HD pathogenesis^21^. However, studies of gene expression changes in HD blood have been inconsistent. Using microarray technology, Borovecki, *et al*.^22^ identified 12 upregulated transcripts, seven of which were also upregulated in brain. However, subsequent studies did not replicate these results^23^,^24^,^25^. Using tag-based serial analysis of gene expression (SAGE), Mastrokolias, *et al*.^25^ found 167 genes differentially expressed by motor score, 40 of which had previously been reported in at least one microarray study.

In the current study we present a transcriptomic analysis of whole blood in human HD using RNA sequencing (RNA-Seq). We studied differential expression of individual gene transcripts and enrichment of differential expression in gene sets in two independent cohorts from Track-HD^26^ and Leiden. We then investigated whether transcriptional changes seen in blood parallel those from previous studies in HD brain. There was significant dysregulation of brain Weighted Gene Correlation Network Analysis (WGCNA) modules in the same direction in blood, as well as significant dysregulation of pathways. Immune gene sets were notably upregulated in both analyses and this signal overlapped with the transcriptional signature of Alzheimer’s disease (AD) brain.

## Results

### No differential expression of individual transcripts in HD whole blood between disease stages or states

Attempting to identify both HD specific and stage-specific changes in gene expression (mRNA) level, we compared premanifest, manifest and control subjects, whilst controlling for age and gender. *Premanifest* gene carriers had a mean total motor score (TMS) of 2 and total functional capacity (TFC) of 13 (Table 1), indicating no substantial motor signs. *Manifest* subjects demonstrated motor abnormalities that were unequivocal signs of HD. No transcripts were significantly differentially expressed (FDR < 0.05) between premanifest and manifest HD in either the Track-HD^26^ or the independently collected Leiden cohort, or when these cohorts were combined (results not shown). As expression changes did not differ significantly between disease stages, all mutant *HTT* gene carriers were combined to increase the analytical power in a comparison of HD and controls. Once again there were no individually significant transcripts in independent or combined cohorts, but the differential expression analysis in the combined cohort is given in Table S1.

### Pathways are dysregulated in HD blood compared with controls

We next asked whether networks of genes with similar functional annotation were dysregulated in HD relative to controls. Pathway annotations were collated from publicly available gene ontology databases to form a set of generic pathways using the same method as the recent HD genome-wide association study (GWAS) of modifiers of age at onset^27^ (see Materials and Methods). The number of pathways significantly dysregulated in both Track-HD and Leiden blood datasets was significantly higher than would be expected by chance (Table 2). Our findings indicate shared biology between the two independent cohorts despite differences in demographic and disease stage; Leiden subjects were on average 7 years older and had correspondingly higher TMS (mean 32 versus 14 in Track-HD) and lower TFC (mean 8 versus 12 in Track-HD). The significance of the overlap was greatly increased in analyses specifying the direction of dysregulation (increased or decreased expression) (Table 2). Therefore, directional analyses were used in the combined dataset as the primary analysis.

**Figure 1:**
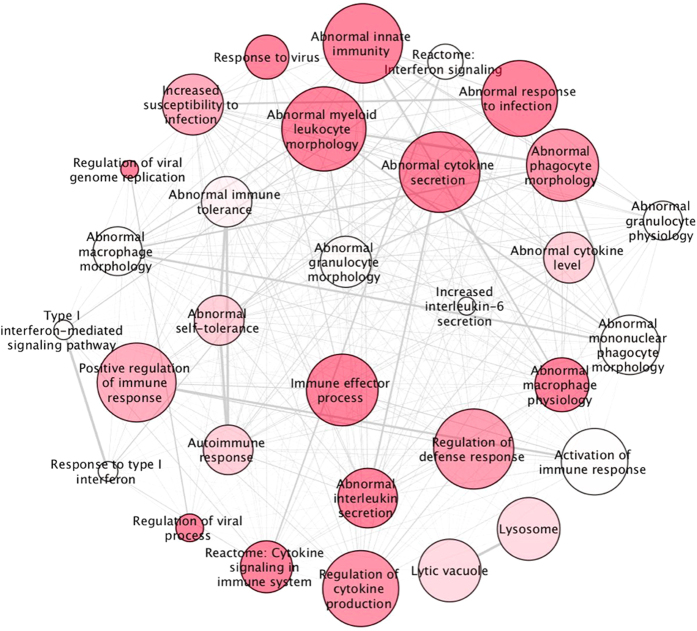
Upregulated pathways in HD versus control blood. Schematic representation of pathways collated from publicly available databases that are significantly upregulated in HD versus controls after correction for multiple testing (q < 0.05). Modules with similar gene content and functional annotation have been consolidated. Nodal shading is inversely proportional to false discovery rate threshold (q value); deep shades have low q values and pale shading is close to the 5% threshold. The weight of connecting lines is proportional to the number of genes shared between pathways.

**Figure 2:**
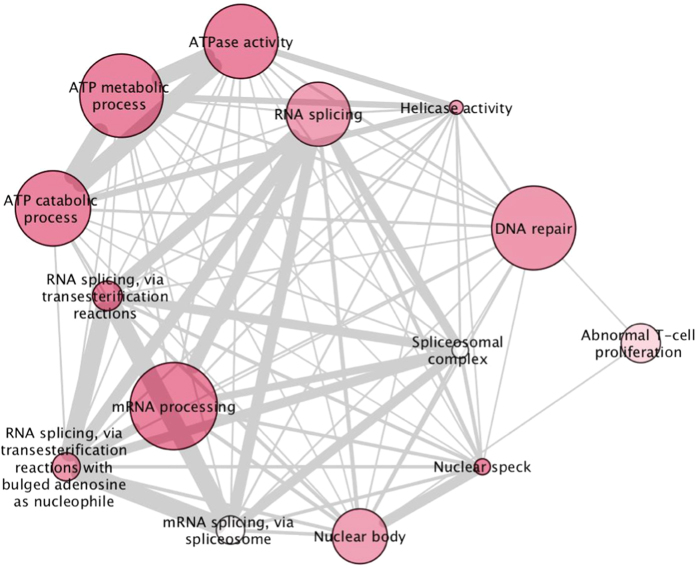
Downregulated pathways in HD versus control blood. Schematic representation of pathways collated from publicly available databases that are significantly downregulated in HD versus controls after correction for multiple testing (q < 0.05). Modules with similar gene content and functional annotation have been consolidated. Nodal shading is inversely proportional to false discovery rate threshold (q value); deep shades have low q values and pale shading is close to the 5% threshold. The weight of connecting lines is proportional to the number of genes shared between pathways.

Gene set enrichment analysis (GSEA), with a false discovery rate (q-value) threshold of q < 0.05 to correct for multiple testing, identified 53 upregulated (Fig. 1 and Table S2) and 14 downregulated pathways (Fig. 2 and Table S3) that are at least nominally significant in both cohorts. Multiple immune-related pathways were upregulated, and RNA processing, ATP metabolism and DNA repair were notably downregulated. The 10 most significant pathways for each direction of dysregulation are given in Table 3 and the full list of significant pathways in Tables S2 and S3. The 10 most dysregulated genes (p < 0.01) from the significantly up or downregulated generic pathways (q < 0.05) are listed in Table S4, and a complete list of genes (p < 0.05) in all nominally significant pathways (p < 0.05) is given in Table S5. Notably, the significantly upregulated pathways contain some of the most differentially expressed transcripts (Table S1), with several more contained in pathways reaching nominal significance (p < 0.05) for dysregulation (Table S5). Genes highlighted by MGI pathways appear distinct from other pathway databases, likely because they are based on knockout studies in mice.

### Pathway dysregulation in HD whole blood overlaps with HD myeloid cells

Through RNA-Seq, Miller, *et al*.^28^ identified transcriptional dysregulation in unstimulated monocytes from HD cases relative to controls. Their GSEA used the same set of generic pathways used here. We found a significant excess of pathways to be significantly (p < 0.05) enriched for dysregulation in both Miller, *et al*.^28^ and the combined TRACK-HD and Leiden whole blood data (Table S6). This overlap was attributable to a significant excess of pathways enriched for upregulation in both datasets. Overlap in downregulated pathways was not significantly larger than expected by chance. Pathways significantly (p < 0.05) enriched for up and downregulation in both myeloid and whole blood are listed in Tables S7 and S8. Pathways that are significantly enriched for upregulation relate mainly to immunity.

### Gene co-expression modules from HD striatum are significantly enriched for dysregulation in HD blood

A limitation of using curated pathways from databases is the incomplete or incorrect annotation. One way to overcome this is to use gene co-expression, because genes that are co-expressed often have related functions. WGCNA identifies clusters (modules) of genes with highly correlated expression, constructing original, unbiased gene co-expression networks based on observed data^29^. HD brain expression modules were generated by Neueder and Bates^30^, who applied WGCNA to Hodges, *et al*.^31^ data and annotated each module that was associated with HD disease status. To further fill the annotation gap and better define functional biological pathways, we generated co-expression modules for control brain from the Braineac^32^ and Gibbs, *et al*.^33^ datasets.

GSEA for brain co-expression modules was applied to our combined Track-HD and Leiden blood expression dataset. Immune- and inflammatory-related brain modules were upregulated in HD blood, and notable downregulated modules included synaptic function, proteasomal degradation, mitochondrial function and transcription. The 10 most significantly up and downregulated modules in the combined dataset that were also nominally significant (p < 0.05) in both independent cohorts are given in Table 4, and the full list of modules nominally significant in both datasets in Table S9. A list of genes from the modules in Table S9 that are themselves nominally significantly dysregulated (p < 0.05) in the combined dataset is given in Table S10. In addition to reinforcing the biological conclusions from our pathway analysis, the significantly dysregulated modules from Table 4 also share genes with the top pathways, as shown in Supplementary Figures S1 and S2. We then investigated whether gene sets that are dysregulated in HD brain^30^ are also disrupted in peripheral blood. Table 5 lists the modules that were significantly dysregulated (after correcting for multiple testing of modules) in both HD brain^30^ and in our combined Track-HD and Leiden blood expression dataset. The direction of dysregulation in brain is shown by the correlation between the module eigengene and HD status (with a positive correlation corresponding to upregulation in the HD brain). Notably, two of the most significantly dysregulated modules in HD caudate^30^ were also significantly dysregulated in the same direction in blood (Table 5), not only in the combined dataset, but in each of the Track-HD and Leiden datasets independently; these being module 48 (CNpos2), which is upregulated in HD, and module 66 (CNneg1), which is downregulated.

The module membership (kME) of a gene is measured by the correlation of its expression with the eigengene, which is representative of all gene expression profiles in the module 34; highly connected ‘hub’ genes have high kME values. Interestingly, among genes in module 48 (CNpos2), the Neueder and Bates^30^ HD caudate module that was also significantly upregulated in blood, there was a significant (p = 7.6 × 10^–4^) correlation between dysregulation p-value in the direction of interest (positive) in HD blood and degree of module membership (kME)^30^. This suggests that highly connected “hub” genes in this module may play a role in transcriptional dysregulation in HD. A similar, although much stronger, effect was noted in caudate^30^. There was no significant correlation in module 66 (CNneg1). Genes in module 48 (CNpos2) that are dysregulated (p < 0.05) in both blood and caudate are shown in Table S11, ranked by their kME value.

### Expression changes in HD blood replicate those in HD prefrontal cortex

Labadorf, *et al*.^35^ identified dysregulated expression of immune and developmental genes in human HD postmortem prefrontal cortex (BA9). Fold changes in expression of individual genes in the combined Track-HD and Leiden data were compared to those observed in Labadorf, *et al*.^35^, and were found to be in the same direction for 8,425 out of the 15,834 genes present in both datasets. This is a highly significant (p < 2.2 × 10^−16^) excess (see Materials and Methods), suggesting some concordance in signal at the individual gene level. Furthermore, a significant excess of generic pathways was found to be significantly (p < 0.05) dysregulated in both datasets, most markedly in the positive (p < 0.001) direction, but also negative (p = 0.028), thus showing an overlap in biological signal. Pathways significantly upregulated in both datasets are mainly related to immune response (Table S12), a pattern also observed in the upregulated brain co-expression modules (Table S13). Pathways downregulated in both datasets are shown in Table S14, with modules in Table S15. Notably, several modules related to the synapse and neuron projection are downregulated in both datasets. The two HD-related caudate modules from Neueder and Bates^30^ that were significantly dysregulated in blood were also significantly dysregulated in the same direction in Labadorf, *et al*.^35^. Module 48 (CNpos2) was significantly upregulated (p < 1 × 10^−16^, Table S13) and module 66 (CNneg1) significantly downregulated (p < 1 × 10^−16^, Table S15), as are several other significant modules from Neueder and Bates^30^.

### Pathways dysregulated in the blood of HD subjects are associated with motor score

We investigated the effect of disease severity by testing for correlation between gene expression and UHDRS total motor score (TMS) in the 112 gene positive Track-HD subjects (Table S16). After correcting for multiple testing, expression of phosphatidylcholine transfer protein (PCTP) was significantly positively correlated with TMS. However, this gene was not found to be significantly correlated with TMS by Mastrokolias *et al*.^25^.

We then tested whether generic pathways that were significantly enriched for upregulated (Table S2) or downregulated (Table S3) genes, also enriched for genes correlated with TMS in the expected direction (Tables S17 and S18) using a similar method to that previously used to test for enrichment of differentially expressed genes. Several immune related pathways were positively correlated with TMS, including MGI:2419, the most significantly dysregulated pathway in HD blood (Table S2). Downregulated pathways that correlated with TMS were related to ATP metabolism and DNA repair.

Similarly, we tested whether modules dysregulated in HD blood relative to controls (Table S9) also correlated with TMS in the expected direction (Table S19). Many modules significantly correlated with TMS, including 68 (CNpos5; p = 5.52 × 10^−7^) and 66 (CNneg1; p = 1.05 × 10^−7^), which were also dysregulated in the HD caudate^30^.

Mastrokolias *et al*.^25^ listed 170 genes significantly associated with TMS, of which 142 passed quality control in our RNA-Seq data. We tested for correlation between these genes and TMS in gene positive subjects from the Track-HD cohort (Table S20). 14 genes were nominally significant (p < 0.05), which is significantly higher than expected by chance (p = 7.89 × 10^−3^). Using the same method as for concordance with Labadorf, *et al*.^35^ (see Materials and Methods), we compared fold changes in expression of individual genes between Track-HD and Mastrokolias *et al*.^25^ Strikingly, 101 genes showed consistent direction of effect, as measured by log(FC), significantly greater than expected by chance (p = 4.78 × 10^−7^). Thus, we conclude that analysis of TMS in the Track-HD cohort broadly supported the associations reported in Mastrokolias *et al*.^25^.

### The Alzheimer’s disease brain transcriptional signature is significantly dysregulated in HD blood

In Alzheimer’s disease, an early inflammatory response involving microglia contributes to pathogenesis^36^,^37^,^38^. Given the upregulation of immune-related gene sets in HD, we next asked whether co-expression modules dysregulated in Alzheimer’s disease (AD) brain were also disrupted in HD blood. Recently the International Genomics of Alzheimer’s Disease Consortium (IGAP) identified four modules from the Gibbs, *et al*.^33^ brain co-expression network that showed enrichment of signal in the GWAS of >70,000 late-onset Alzheimer’s disease (LOAD) and control subjects^39^. These four modules, each derived from a different brain region, are all involved in the immune response and were all significantly upregulated in our combined HD blood dataset (Table S21). Module 56, derived from pontine data, was also significantly enriched in both Track-HD and Leiden datasets independently. IGAP identified 151 genes that were present in two or more of these modules and showed the most significant enrichment with LOAD GWAS signal^39^. These 151 genes were also significantly enriched for upregulation in the combined HD blood dataset (p = 2.50 × 10^−4^).

Zhang, *et al*.^40^ identified co-expression modules that were differentially connected between LOAD and controls. Ten of these were also significantly enriched for upregulation in our HD blood expression dataset (Table S22) after correction for multiple testing (q < 0.05), with their most significant module, *yellow*, being particularly highly enriched (combined Track-HD and Leiden p < 1 × 10^−16^). Notably, this module has immune and microglia-specific functions^40^. This enrichment for modules from the IGAP GWAS^39^ and Zhang, *et al*.^40^ in the HD blood transcriptome suggests a shared immune-related mechanism between different neurodegenerative diseases, at least including HD and Alzheimer’s disease.

## Discussion

HD research has focused on the brain as the most conspicuous clinical features can be clearly linked to progressive degeneration of specific brain regions^4^,^5^. However, HD is a systemic condition with peripheral expression of mutant huntingtin directly driving abnormalities such as immune dysfunction, metabolic derangement and transcriptional dysregulation that contribute to onset, progression, quality of life and mortality^5^,^7^.

We conducted RNA-Seq of whole blood in two independent cohorts of HD patients. Using gene set enrichment analysis (GSEA) with publicly-available pathway databases and WGCNA modules from HD and control brain datasets, we identified dysregulated genes and gene sets in blood that replicated in both independent cohorts and correlated with clinical motor signs (TMS). These correspond to the most significantly dysregulated modules in caudate nucleus, the most prominently affected region in HD brain. This suggests mutant huntingtin drives a common pathogenic signature in both blood and brain.

RNA-Seq more comprehensively and accurately quantifies mRNA than hybridisation-based microarrays or tag-based methods^41^. Expression of phosphatidylcholine transfer protein (PCTP) significantly correlated with TMS (Table S16). This protein transports phospholipids across intracellular membranes, which is of interest given the upregulation of lipid metabolic modules identified above (Tables 4 and 5) and increasing evidence for a pathological interaction between mutant huntingtin and membrane phospholipids^42^. However, PCTP was not significantly correlated with TMS in Mastrokolias *et al*.^25^. It is perhaps unsurprising that there was limited differential expression of individual transcripts by disease state (Table S1) or severity in either the independent or combined cohorts; the major cell types known to contribute to symptoms are not present in blood and the haematogenous cells known to be dysfunctional in HD, such as monocytes and macrophages^9^,^43^, constitute only a small proportion of circulating cells^44^. The variation of gene expression in blood with age, gender, cell type and time of day is also likely to contribute^44^,^45^. Our results are consistent with previous studies that have shown weak correlation at the transcript level between blood and brain^46^.

Despite these limitations, gene set enrichment analysis identified significantly overlapping dysregulated pathways in the Track-HD and Leiden HD blood datasets, even though they differed in age and disease severity. Thus, through grouping transcripts into biologically relevant pathways and co-expressed transcripts, we could highlight areas of dysfunctional biology in HD. The observed upregulation of immune-related pathways is consistent with that previously identified in transcriptional and functional studies^5^,^7^,^25^. HD patients are known to have immune dysfunction, both in the central nervous system (CNS) with microglial activation^8^, and peripherally with elevated proinflammatory cytokines in premanifest carriers up to 16 years before predicted onset^9^,^43^. The migration of phagocytic cells is impaired in HD^10^,^11^ and patient-derived monocytes are hyperactive on stimulation, an effect reduced by HTT lowering^9^. Modulation of the peripheral immune system with a type 2 cannabinoid receptor (CB2) agonist^47^ or bone marrow transplantation^48^ can increase lifespan and reduce motor deficits and synaptic loss in HD mouse models.

RNA processing pathways were downregulated, which is congruent with known decreases in miRNAs and altered expression of key miRNA processing enzymes in HD^49^. Consistent with the downregulation of pathways involved in energy metabolism that we observe, mitochondrial ATP is known to be reduced in HD brain^50^ and blood^51^, and *PGC-1α*, a member of the dysregulated *ATP metabolic process* pathway (Tables 3, S14 and S18), is a key protective regulator of mitochondrial genes that is repressed HD mouse models^52^,^53^. Downregulation of genes involved in DNA repair is likely to be relevant to somatic expansion that may influence disease onset and progression^54^. The signature of pathway dysregulation we identified in HD whole blood correlates with TMS in HD subjects from Track-HD. It also significantly overlaps with that recently found in unstimulated HD monocytes^28^. This enrichment was driven primarily by upregulation of immune pathways, as might be expected given that Miller, *et al*.^28^ isolated myeloid cells.

To overcome the annotation gap commonly observed with publicly-derived pathway databases and to investigate whether gene expression changes from HD brain are also present in blood, we performed GSEA using brain co-expression networks derived from HD^30^ and control^32^,^33^ subjects. Several HD brain modules were significantly dysregulated in HD blood, suggesting a common signature of transcriptional dysregulation between blood and brain.

Brain modules upregulated in blood were enriched for immune-related genes, confirming the results of our pathway analysis. Strikingly, two of the modules most significantly dysregulated in HD caudate, 48 (CNpos2) and 66 (CNneg1), were also significantly dysregulated in the same direction in both independent blood datasets. Compared with other brain regions, the caudate has the largest number of expression changes and the highest correlation with HD^30^. Module 48 (CNpos2), the second most significantly upregulated module in caudate, is enriched for transcriptional regulators, chromatin modifiers and genes involved in mRNA processing^30^. We also find this module to be significantly enriched for immune response genes, giving further support to the pathway results. Module 66 (CNneg1), the most significantly downregulated module in caudate, contains genes involved in neuronal function, particularly synaptic function and plasticity, and ion channels. Around half of its hub genes are implicated in synaptic function and all were significantly downregulated in Hodges, *et al*.^31^. Though synapses are not present in blood, synaptic genes may be dysregulated in circulating cells without significant pathogenic impact, or alternatively they may serve distinct functions in blood cells. Indeed, Cai, *et al*.^46^ found that the synaptic module was well preserved between brain and blood. We also found that gene expression and pathway dysregulation from HD prefrontal cortex^35^ was replicated in HD blood. The high degree of replication increases confidence in the shared signal between blood and brain. A significant proportion of the modules dysregulated in HD blood correlated with TMS.

Our demonstration of a transcriptional signature common to both HD blood and brain supports the use of blood cells to study aspects of HD biology. HD model systems, such as mice, only recapitulate aspects of disease and must be compared to the relevant data in human tissue^55^,^56^. Access to brain tissue is very limited and tends to be from post-mortem subjects with advanced disease, which affects RNA integrity^19^,^20^. Blood, by contrast, is readily available and can be obtained longitudinally from HD subjects. Recently, Mina, *et al*.^57^ performed WGCNA on the Leiden blood sample, finding modules related to immune response that were associated with TFC and TMS. Furthermore, by comparing biological annotations of their HD blood modules with those they derived from Hodges, *et al*.^31^ brain expression data, they showed a common signature between blood and caudate related to immune response. These analyses, using different methodology to ours, lend further support to our conclusions.

In AD, amyloid plaques are surrounded by chronically activated microglia^36^,^37^ and GWA studies have identified immune-related genes as risk factors for LOAD^58^. Recently Hong, *et al*.^38^ showed that early in the disease process, before plaque formation, microglia and complement activation drive synaptic loss, a process that may reflect reactivation of developmental synaptic pruning^59^. In HD blood we found significant upregulation of immune modules associated with AD in the IGAP GWAS^39^, a subset of genes with shared membership of several of these modules, and the most significant immune and microglia-related modules from Zhang, *et al*.^40^. In a co-expression network generated from prefrontal cortex of 194 HD patients, Zhang, *et al*.^40^ found that their most significant immune and microglia module was well conserved, though was not significantly dysregulated in HD and did not correlate with CAG repeat length. This may be because cortex shows less severe pathology and transcriptional dysregulation than caudate^21^. Overlapping immune upregulation in HD and AD suggests these two distinct neurodegenerative diseases share some common pathogenic mechanisms, including macrophage function^38^. Improved understanding of these mechanisms may open the way to therapeutic targets in these currently incurable diseases.

## Materials and methods

All experiments we performed in accordance with the Declaration of Helsinki and approved by the University College London (UCL)/UCL Hospitals Joint Research Ethics Committee and the LUMC IRB. Peripheral blood samples were donated by genetically-diagnosed HD patients and controls, and all subjects provided informed written consent.

### Cohorts

The Track-HD cohort consisted of 54 premanifest gene carriers, 63 manifest HD subjects and 23 controls. These were a representative sample from the Track-HD study (Table 1), preselected to assure a wide range of disease risk and severity. Control subjects were age and gender matched to individuals in the premanifest and manifest groups, and selected from spouses or partners to ensure consistency of environments. Track-HD enrolled participants at four study sites in London (UK), Paris (France), Leiden (Netherlands), and Vancouver (BC, Canada)^26^. *Manifest* subjects demonstrated motor abnormalities that were unequivocal signs of HD, as evidenced by total motor scores (TMS) over 5 on the Unified Huntington’s Disease Rating Scale (UHDRS). *Premanifest* gene carriers had a burden of pathology score (age x [CAG – 36.5))^60^ greater than 250, and a TMS of 5 or lower and a diagnostic confidence score (DCS) less than 4 on the UHDRS^61^, indicating no substantial motor signs^26^. Age and clinical scores considered for the analysis were at time of blood collection.

The Leiden cohort^25^ consisted of 18 premanifest gene carriers, 56 manifest HD subjects and 27 age and gender-matched controls. Motor onset was determined by an experienced neurologist using the same UHDRS standard as in TRACK-HD. All premanifest carriers showed no substantial motor signs, with a TMS of 5 or less and a UHDRS diagnostic confidence level less than 4. All controls were free of known medical conditions. Blood sample collection and analysis methods, described below, were identical for the two cohorts.

### Sample collection

Whole blood was collected in two PAXGene Blood RNA tubes (PreAnalytix, Qiagen/BD Company) per subject, and immediately placed upright at room temperature. They were checked at 5 hours for incomplete mixing or separation, and any showing separation were remixed with a further 10 inversions. Tubes were stored overnight at −20 °C and transferred to −80 °C the following morning. They were sent on dry ice to Biorep within 30 days.

### RNA preparation

Total RNA extraction was performed using the PAXGene Blood RNA kit (catalog N. 762174; PreAnalytix, Qiagen/BD Company), following the supplier’s instructions. Each solution in the kit was divided into aliquots to process batches of 12 samples. Replicate tubes for each subject were processed on different days. RNA was stored at −80 °C before proceeding with the quality measurements and further use. RNA was collected by centrifugation, washing with 70% ethanol, and resuspended in buffer. Quality measurements of total RNA were made using spectrophotometric analysis (Nanodrop), 260/280 ratio denaturing agarose gel, and the RNA 6000 Nano kit for the Agilent Bioanalyzer (catalog N. 5067-1511, Agilent Technologies). Samples were globin reduced using the GLOBINclear^TM^ method (catalog N. AM1980, ThermoFisher Scientific). Quality control measures were made on globin-reduced samples on the Bioanalyzer RNA 6000 Nano kit (Catalog N. 5067-1511, Agilent Technologies).

### Sequencing

Indexed cDNA sequencing libraries were prepared using the TruSeq^TM^ Poly-A mRNA method (Illumina). In short, poly-A mRNA transcripts were captured from total RNA using poly-T beads and cDNA generated using random hexamer priming^62^. Paired-end sequencing of indexed cDNA libraries on a HiSeq 2500 generated at least 50 M reads per sample. Sequencing was performed using SBS and cluster kits from Illumina. Indexed samples were demultiplexed and FASTQ files were generated.

### Quality control

Sequencing failed for six Track-HD samples, including four premanifest, one manifest and one control subject. Quality control analysis was performed using the RNA-SeQC package^63^, ensuring measures including rRNA rate, mapping rate, concordance mapping rate and uniqueness rate were within acceptable ranges. Globin depletion was checked by inspecting read counts mapped to HBB, HBA1 and HBA2, confirming they made up less than 2% of reads for all samples. Four Track-HD and six Leiden samples failed quality control for duplication rate over 75%, GC bias or 5’ bias, and were removed, leaving 48 premanifest, 61 manifest and 21 control subjects in the Track-HD cohort and 15 premanifest, 54 manifest and 26 control subjects in the Leiden cohort.

### Gene expression analysis

RNA-Seq data were aligned to the human reference genome hg19 using TopHat2^64^. Read counts were summarised using HTSeq, keeping any duplicates and using the Ensembl transcript/gene database (http://www.ensembl.org/info/data/ftp/index.html, obtained in gtf format, genome build GRCh38.3, gene build updated in June 2015). To remove residual batch effects the R package svaseq was used^65^. Using the cleaned count data, differential expression analysis was conducted using the R package DESeq2^66^. Outlier counts were removed using a Cooks distance cutoff of 5 in DESeq2. After filtering by the mean of normalised counts, 18,257 transcripts were detected. Age and gender were used as covariates in the analysis.

### Pathway analysis

Enrichment of differential expression among gene sets corresponding to biological hypotheses (pathways) was tested using the Gene Set Enrichment Analysis (GSEA) method^67^. Rather than defining a list of significant genes, GSEA ranks all genes in order of their differential expression statistic, and tests whether the genes in a particular gene set have a higher rank overall than would be expected by chance. The analysis is weighted by the differential expression statistic, thus giving more weight to more significant genes. Significance of enrichment was obtained by randomly permuting gene-wide association statistics among genes. One-sided p-values were calculated separately for differential upregulation and downregulation of expression in HD, and these were then converted into the corresponding chi-square statistic for use in the GSEA analysis. To avoid making a priori assumptions, we collated a large pathway set from publicly available pathway databases, including Gene Ontology (GO)^68^, Kyoto Encyclopedia of Genes and Genomes (KEGG)^69^, Mouse Genome Informatics (MGI)^70^, PANTHER^71^, BioCarta^72^, REACTOME^73^ and NCI^74^. This resulted in a total of 14,706 functional pathways, many with overlapping members, containing between 3 and 500 genes. To correct for multiple testing of pathways we converted the GSEA p-values into q-values^75^, which can be interpreted as the minimum false discovery rate at which that q-value would be counted as significant.

### Gene co-expression networks

Weaknesses of relying on public databases to provide pathways for analysis include their restriction to prior biological knowledge and the poor annotation of many genes. To overcome this annotation gap, we also tested the following sets of gene co-expression modules for enrichment of dysregulation:

1. The set of 124 HD brain expression modules derived by Neueder and Bates^30^, who applied weighted gene correlation network analysis (WGCNA)^34^ to the Hodges, *et al*.^31^ microarray brain expression data set of 44 human HD and 36 matched control brains. They generated networks for four brain regions; the caudate nucleus (CN), BA4 region of the frontal cortex, which has motor function (FC-BA4), BA9 region of the frontal cortex, involved in association and cognitive functions (FC-BA9), and cerebellum (CB).
2. A set of 117 co-expression modules derived from the Gibbs, *et al*.^33^ dataset, comprising microarray expression data from 150 control individuals measured in four brain regions: cerebellum (CB), frontal cortex (FC), caudal pons (Pons) and temporal cortex (TCTX). Modules were generated using WGCNA as described in ref. 39.
3. We generated a set of 213 co-expression modules from Braineac^32^, which consists of microarray expression data for 12 brain regions from 134 control brains; occipital cortex, frontal cortex, temporal cortex, hippocampus, intralobular white matter, cerebellar cortex, thalamus, putamen, substantia nigra, and medulla (inferior olivary nucleus). For each brain region, the array data was normalised in the R statistical-programming environment using the RMA algorithm^76^. Principal Component Analysis (PCA) and hierarchical clustering were used to identify single outlier arrays for removal. In addition, small outlier clusters (<6 arrays) that were distinct from most of the other arrays were removed (i.e. small clusters appearing at the top of the dendrogram). Once outlier arrays were removed, the arrays were re-normalized and inspected again and re-processed if necessary until a homogenous dataset was produced. WGCNA was performed using the R package to derive modules^34^. Multiple probesets of the same gene were collapsed to a single value using the collapseRows() function, using default settings and based on gene annotation provided by Affymetrix^77^. Scale independence and mean connectivity were plotted to derive a soft threshold power of 6. Networks were unsigned.
4. The set of 111 co-expression modules from Zhang, *et al*.^40^, generated using microarray expression data on 1,647 postmortem samples from three brain regions of late-onset Alzheimer’s disease (LOAD) and control subjects; prefrontal cortex (BA9), primary visual cortex (BA17), and cerebellum.

### Concordance of fold change in gene expression between datasets

Labadorf, *et al*.^35^ analysed the transcriptome of human postmortem prefrontal cortex Brodmann area 9 (BA9) from 20 HD subjects and 49 controls using next-generation high throughput sequencing, identifying dysregulation of immune and developmental genes. Of the 15,834 genes common to both the combined Track-HD and Leiden blood dataset and the Labadorf, *et al*.^35^ prefrontal cortex dataset, 8447 had a fold change >1 (i.e. upregulated) in blood and 7860 in cortex. Thus, if fold changes in the two datasets were assumed to be unrelated, the expected probability that a gene would show concordant fold change is equal to ((8447/15834)x(7860/15834)) + ((7387/15834)x(7974/15834)) = 0.4997. The number of genes with concordant fold change in the absence of a relationship between the datasets is thus distributed as a binomial (15834, 0.4997) distribution. In the actual data, 8425 genes were observed to have concordant direction of fold change, significantly higher than the number expected by chance (7912).

We used a similar method to test for concordance of fold change in genes between the Track-HD and Mastrokolias *et al*. datasets.

### Data availability

All data is deposited at the European Genome-phenome Archive (EGA) and accessible through the authors or the NeurOmics consortium.

## Additional Information

**How to cite this article**: Hensman Moss, D. J. *et al*. Huntington’s disease blood and brain show a common gene expression pattern and share an immune signature with Alzheimer’s disease. *Sci. Rep.* **7**, 44849; doi: 10.1038/srep44849 (2017).

**Publisher's note:** Springer Nature remains neutral with regard to jurisdictional claims in published maps and institutional affiliations.

## Supplementary Material

Supplementary Figures

Supplementary Tables

## Figures and Tables

**Table 1:** Track-HD and Leiden cohorts for RNA-Seq analysis.

Cohort	Group	n	Mean age, y ± SD (range)	Gender (male/female)	Mean (CAG)n length ± SD (range)	Mean TMS ± SD (range)	Mean TFC ± SD (range)
Track-HD	*Premanifest*	*50*	*42* ± *9 (22*–*64*)	*24/26*	*43* ± *3 (39*–*52*)	*2* ± *2 (0*–*8*)	*13* ± *0 (12*–*13*)
	*Manifest*	*62*	*48* ± *10 (23*–*64*)	*26/36*	*44* ± *3 (39*–*59*)	*23* ± *11 (5*–*45*)	*11* ± *2 (7*–*13*)
	HD	112	46 ± 10 (22–64)	50/62	44 ± 3 (39–59)	14 ± 13 (0–45)	12 ± 2 (7–13)
	Control	22	45 ± 5 (34–53)	9/13	—	—	—
Leiden	*Premanifest*	*18*	*46* ± *10 (29*–*63*)	*5/13*	*42* ± *2 (39*–*47*)	*3* ± *2 (0*–*5*)	*12* ± *1 (10*–*13*)
	*Manifest*	*56*	*55* ± *11 (35*–*79*)	*29/27*	*44* ± *3 (39*–*53*)	*42* ± *30 (6*–*102*)	*7* ± *5 (0*–*13*)
	HD	74	53 ± 11 (29–79)	34/40	44 ± 3 (39–53)	32 ± 31 (0–102)	8 ± 5 (0–13)
	Control	27	43 ± 11 (26–65)	13/14	—	—	—
Combined	HD	186	48 ± 11 (22–79)	84/102	44 ± 3 (39–59)	21 ± 24 (0–102)	10 ± 4 (0–13)
	Control	49	44 ± 9 (26–65)	22/27	—	—	—

*Manifest* subjects demonstrated motor abnormalities that were unequivocal signs of HD. *Premanifest* gene carriers had a total motor score of 5 or lower and a diagnostic confidence score (DCS) less than 4 on the UHDRS, indicating no substantial motor signs. The *HD* group consists of the combined *premanifest* and *manifest* subjects. Controls were matched for age and gender. Age and clinical scores considered for the analysis were at time of blood collection. SD – standard deviation; TFC – Total Functional Capacity; TMS – Total Motor Score.

**Table 2:** Overlap analysis of Track-HD and Leiden cohorts shows that a significant excess of pathways are associated with HD (p < 0.05) in both datasets.

Reference dataset	Comparison dataset	Direction of dysregulation in HD	Number of pathways significant in both datasets (p value)		
			Generic pathways	HD brain modules	Control brain modules
Leiden	Track-HD	Nondirectional	69 (4.6E-02)	—	—
		Downregulated	130 (<1.0E-03)	4 (1.1E-01)	24 (<1.0E-03)
		Upregulated	219 (<1.0E-03)	9 (<1.0E-03)	23 (<1.0E-03)
Track-HD	Leiden	Nondirectional	69 (1.4E-01)	—	—
		Downregulated	130 (1.7E-02)	4 (3.5E-02)	24 (<1.0E-03)
		Upregulated	217 (<1.0E-03)	10 (<1.0E-03)	21 (<1.0E-03)

Significance of overlap is greatest when directionality is taken into account. There is an excess of significantly enriched pathways and modules in the reference dataset conditional on the pathway being enriched (p < 0.05) in the comparison dataset. The *generic pathways* gene set is collated from publicly-available databases including GO and KEGG. *HD brain modules* are derived from Neueder and Bates^30^. *Control brain modules* are from the Braineac^32^ and Gibbs, *et al*.^33^ expression datasets.

**Table 3:** The 10 most significantly up and downregulated ‘generic’ pathways in HD versus control blood GSEA.

Direction of dysregulation in HD	Pathway	Number of dysregulated genes	p (combined)	q (combined)	p (Track-HD)	p (Leiden)	Description
Upregulated	MGI: 2419	434	3.03E-10	4.32E-06	5.10E-05	3.01E-05	Abnormal Innate Immunity
	MGI: 3009	432	5.78E-09	4.13E-05	5.96E-06	1.65E-04	Abnormal Cytokine Secretion
	GO: 50792	117	2.59E-08	1.23E-04	1.12E-02	7.24E-05	Regulation Of Viral Process
	GO: 9615	208	1.22E-07	4.36E-04	3.06E-02	5.34E-06	Response To Virus
	MGI: 2451	278	1.68E-07	4.80E-04	1.26E-02	9.51E-06	Abnormal Macrophage Physiology
	GO: 19221	308	2.38E-07	5.45E-04	4.60E-05	1.71E-04	Cytokine-Mediated Signaling Pathway
	GO: 2252	365	3.10E-07	5.45E-04	7.01E-03	1.14E-04	Immune Effector Process
	MGI: 5025	406	3.44E-07	5.45E-04	5.91E-05	2.02E-04	Abnormal Response To Infection
	MGI: 1793	372	4.33E-07	5.82E-04	5.93E-05	2.42E-04	Altered Susceptibility To Infection
	MGI: 8568	305	4.49E-07	5.82E-04	4.79E-05	6.25E-05	Abnormal Interleukin Secretion
Downregulated	GO: 8380	282	5.22E-08	7.45E-04	4.25E-05	7.24E-05	RNA splicing
	GO: 6397	359	2.38E-07	1.70E-03	1.48E-04	4.14E-04	mRNA processing
	GO: 16887	329	1.37E-06	5.48E-03	1.96E-04	3.34E-02	ATPase activity
	GO: 6200	333	1.54E-06	5.48E-03	2.42E-04	3.36E-02	ATP catabolic process
	GO: 46034	361	5.36E-06	1.53E-02	1.74E-04	4.45E-02	ATP metabolic process
	GO: 16607	144	9.06E-06	2.15E-02	4.68E-04	4.61E-03	Nuclear speck
	GO: 6281	356	1.66E-05	2.75E-02	2.00E-03	1.18E-04	DNA repair
	GO: 16604	271	2.08E-05	2.75E-02	5.59E-03	2.46E-03	Nuclear Body
	GO: 4386	135	2.12E-05	2.75E-02	2.83E-02	4.81E-02	Helicase Activity
	GO: 375	184	2.40E-05	2.86E-02	1.14E-03	2.05E-03	RNA splicing, via transesterification reactions

A total of 14,706 Generic pathways, each containing between 3 and 500 genes, were collated from publicly-available databases including GO and KEGG. Pathways are significantly dysregulated after multiple testing correction (q < 0.05). Enrichment p values in the current study for the Track-HD, Leiden and combined datasets are shown.

**Table 4:** The 10 most significantly up and downregulated WGCNA brain expression modules in HD versus control blood.

Direction of dysregulation in HD	Brain expression gene set	Module	Brain region	Annotation	Number of genes	p (Combined)	p (Track-HD)	p (Leiden)
Upregulated	HD	111	FC BA9	Immune response	514	7.8E-12	1.3E-04	7.5E-05
	HD	69 (FC4pos1)	FC BA4	Inflammatory response	712	3.8E-08	3.1E-05	1.3E-03
	Control (Braineac)	712	TCTX	Inflammatory response	213	1.4E-07	3.4E-05	8.1E-04
	HD	48 (CNpos2)*	CN	Lipid metabolism/regulation of transcription	1785	2.0E-07	3.9E-03	6.3E-03
	Control (Braineac)	110	FCTX	Inflammatory response	173	8.9E-07	1.0E-03	2.5E-03
	Control (Braineac)	909	White Matter	Activation of immune response	265	2.1E-06	1.2E-03	2.5E-02
	Control (Braineac)	610	Substantia Nigra	Inflammatory response	178	1.2E-05	8.6E-04	5.6E-04
	Control (Braineac)	811	Thalamus	Inflammatory response	142	1.6E-05	3.9E-03	2.9E-03
	Control (Gibbs)	56	Pons	Lipoprotein/ immune response /GTPase regulator activity	207	2.0E-05	2.4E-04	4.2E-02
	Control (Braineac)	911	White Matter	Inflammatory response	159	3.0E-05	8.4E-04	1.4E-02
Downregulated	Control (Gibbs)	22	CB	Pro-rich region	831	1.8E-08	2.5E-03	2.1E-02
	Control (Gibbs)	28	FC	Intra-cellular transport/mitochondrion	3178	2.1E-08	6.3E-04	7.7E-05
	Control (Braineac)	304	Medulla	mRNA metabolic process	1811	2.9E-08	5.0E-15	4.0E-02
	HD	66 (CNneg1)*	CN	Synapse/ion channels	2645	2.7E-07	1.5E-04	2.1E-02
	Control (Braineac)	804	Thalamus	Regulation of cell morphogenesis	857	1.3E-06	4.0E-02	4.1E-04
	Control (Braineac)	522	Putamen	Regulation of RNA splicing	64	4.4E-06	6.3E-03	2.7E-04
	Control (Gibbs)	74	Pons	Transcription/acetylation/protein transport	1183	9.2E-06	3.9E-08	7.4E-04
	Control (Braineac)	702	TCTX	Antigen processing: ubiquitination and proteasome degradation	4602	3.9E-04	1.2E-03	2.5E-02
	Control (Gibbs)	48	FC	Transcription corepressor/cell morphogenesis	648	4.7E-04	7.8E-03	2.1E-02
	Control (Braineac)	202	Hippocampus	Mitochondrial membrane	2737	4.8E-04	1.2E-07	1.5E-02

All modules in this table are significantly dysregulated after correction for multiple testing (q < 0.05) in the combined blood sample. *HD brain* modules were defined by Neueder and Bates^30^, and *Control brain* modules were generated from Braineac^32^ and Gibbs, *et al*.^33^. Neueder and Bates^30^ module identifiers are given in brackets where available. *Denotes the caudate modules that were highly positively and negatively Neueder and Bates^30^. *CN* – caudate nucleus; *FC* – frontal cortex; *FC BA4* – BA4 region of the frontal cortex; *FC BA9* – BA9 region of the frontal cortex; *CB* – cerebellum; *TCTX* – temporal cortex.

**Table 5:** Brain expression modules significantly dysregulated both in HD brain and HD blood.

Module number	Brain Region	Module name	Number of genes	p (combined)	p (TRACK)	p (Leiden)	cor (HD brain)	p (HD brain)	Description
69	FC_BA4	FC4pos1	712	3.77E-08	3.05E-05	1.32E-03	0.610	3.77E-03	Inflammatory response
48	CN	CNpos2	1785	2.03E-07	3.85E-03	6.33E-03	0.724	2.21E-11	Lipid metabolism/regulation of transcription
64	CN	CNpos6	114	3.13E-04	1.18E-02	3.80E-02	0.463	2.28E-04	Inflammatory response
66	CN	CNneg1	2644	2.71E-07	1.51E-04	2.13E-02	−0.800	6.03E-15	Synapse

All modules in this table are significantly dysregulated after correction for multiple testing (q < 0.05) in the combined blood sample, and are nominally significantly dysregulated (p < 0.05) in both Track-HD and Leiden datasets separately. *Cor(HD brain)* – the correlation between module eigengene and HD status observed by Neueder and Bates^30^ in brain expression data, with a positive correlation corresponding to upregulation in HD. *p(HD brain*) is the p-value for that correlation (corrected for multiple testing of modules).
