# Supplementary material for: Huntington’s disease blood and brain show a common gene expression pattern and share an immune signature with Alzheimer’s disease
Source: Sci Rep. 2017 Mar 21;7:44849. doi: 10.1038/srep44849 (PMC5359597; doi:10.1038/srep44849)
Supplement: Supplementary Figures [file srep44849-s1.pdf]

Supplementary information

Title

Huntington's disease blood and brain show a common gene expression pattern and share an immune signature with Alzheimer's disease

Authors

Davina J Hensman Moss [+1], Michael D Flower [+1], Kitty K Lo[2], James RC Miller[1], Gert-Jan B van Ommen[3], Peter AC 't Hoen[3], Timothy C Stone[4], Amelia Guinee[5], Douglas R Langbehn[6], Lesley Jones[4], Vincent Plagnol[2], Willeke MC van Roon-Mom[3], Peter Holmans*[4], Sarah J Tabrizi*[1]

[+] These authors contributed equally

* These authors contributed equally

Supplementary figures

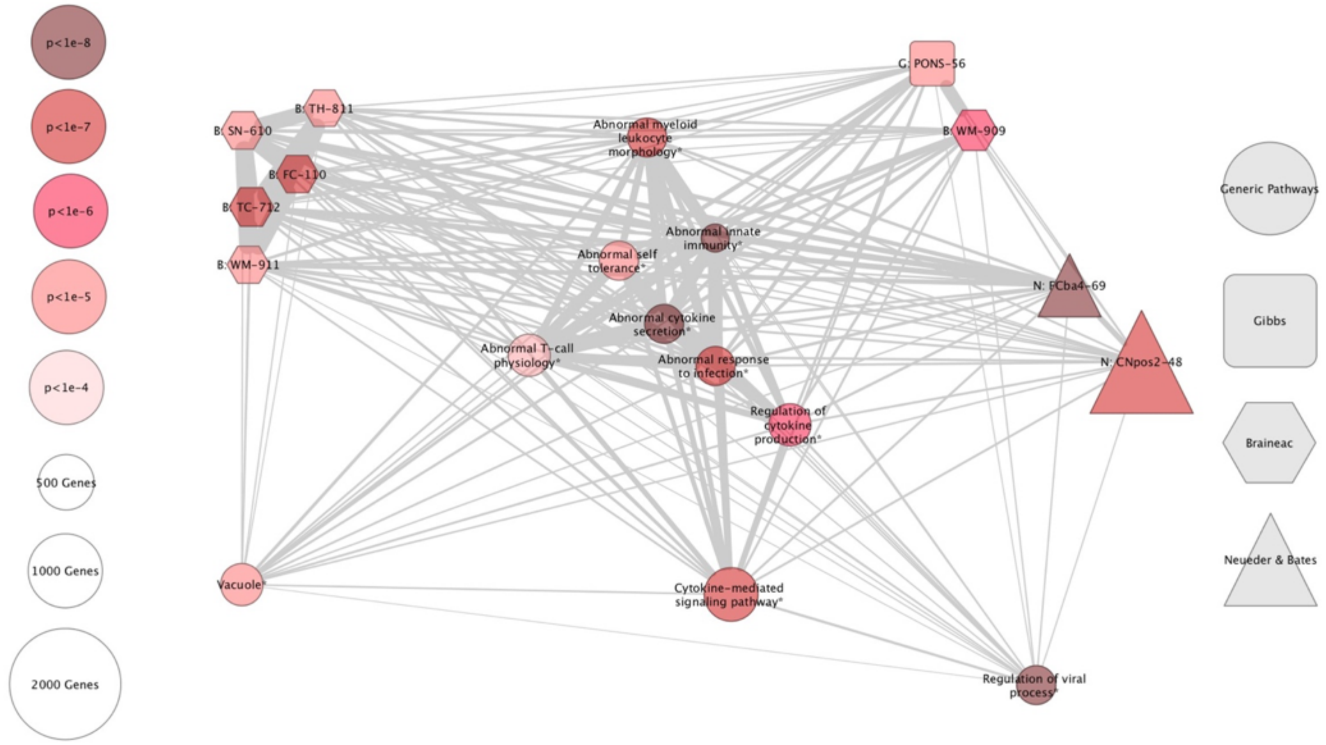

Figure S1. Network diagram of the relationship between significantly (q<0.05) upregulated gene modules (Table 4) and generic biological pathways (Table S2) based on shared gene membership. The thickness of the edges corresponds to the proportion of overlap from the smaller term to the larger (overlap coefficient). Intensity of shading indicates p-value (darker colours have lower p-values), node size indicates size of gene content, node shape indicates origin of data (modules or pathways). For clarity, biological pathways with similar gene content are grouped together, as described in Supplementary Table S18, and the shading reflects the most significant pathway in the group. Nodes are arranged such that the distance between them reflects similarity in gene content. Diagram rendered in Cytoscape.

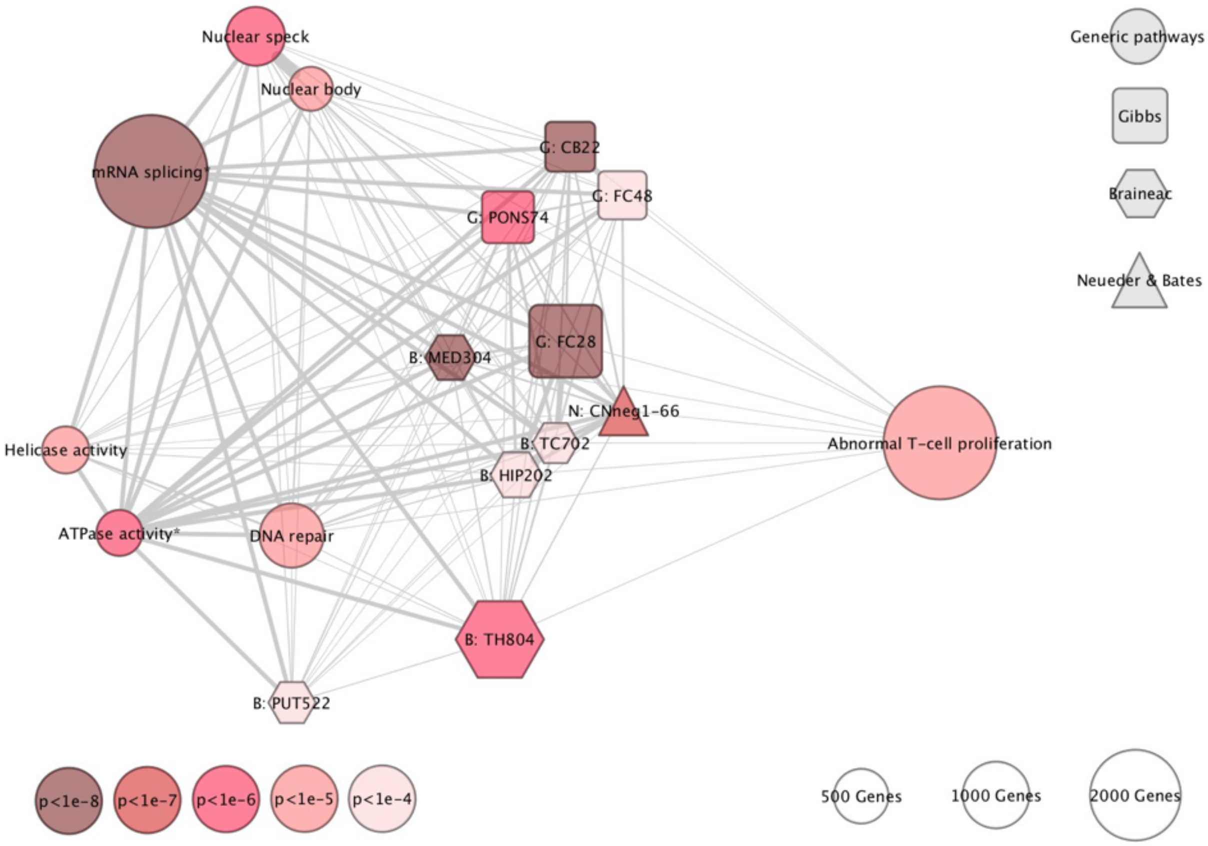

Figure S2. Network diagram of the relationship between significantly (q<0.05) downregulated gene modules (Table 4) and generic biological pathways (Table S3) based on shared gene membership. The thickness of the edges corresponds to the proportion of overlap from the smaller term to the larger (overlap coefficient). Intensity of shading indicates p-value (darker colours have lower p-values), node size indicates size of gene content, node shape indicates origin of data (modules or pathways). For clarity, biological pathways with similar gene content are grouped together, as described in Supplementary Table S19, and the shading reflects the most significant pathway in the group. Nodes are arranged such that the distance between them reflects similarity in gene content. Diagram rendered in Cytoscape.

Supplementary tables

Table S1. Differential expression analysis in HD (premanifest and manifest combined) versus controls for the combined Track-HD and Leiden cohorts. *p (diffexp)* – p value for differential expression between HD and

controls; *q (diffexp)* – q value shows correction for multiple testing in the combined dataset; *Log2(FC)* – log2 of the ratio of the mean counts in HD and controls.

Table S2. All significantly upregulated generic pathways ($p < 0.05$) in both Track-HD and Leiden datasets from HD versus control blood GSEA. A total of 14,706 generic pathways, each containing between 3 and 500 genes, were collated publicly-available databases including GO and KEGG. q values show correction for multiple testing in the combined dataset. Enrichment p values for Track-HD, Leiden and combined analyses are given.

Table S3. All significantly downregulated generic pathways ($p<0.05$) in both Track-HD and Leiden datasets from HD versus control blood GSEA. A total of 14,706 generic pathways, each containing between 3 and 500 genes, were collated publicly-available databases including GO and KEGG. q values show correction for multiple testing in the combined dataset. Enrichment p values for Track-HD, Leiden and combined analyses are given.

Table S4. The 10 most significantly dysregulated genes ($p<0.01$) in up or downregulated generic pathways ($q<0.05$). *p (Comb/Track-HD/Leiden*) – p value for differential expression between HD and controls in the combined, Track-HD or Leiden datasets; *Log2FC* – log2 of the ratio of mean counts in HD and controls.

Table S5. All significantly dysregulated genes ($p<0.05$) from generic pathways that were dysregulated (up or down) in HD blood. *p (Comb/Track-HD/Leiden*) – p value for differential expression between HD and controls in the combined, Track-HD or Leiden datasets; *Log2FC* – log2 of the ratio of the mean counts in HD and controls.

Table S6. Number of pathways nominally significantly enriched (uncorrected p<0.05) in both the combined Track-HD/Leiden blood dataset and the unstimulated myeloid data of Miller, et al. [1]. The p-value measures whether there is an excess of significantly enriched pathways in the blood dataset conditional on the pathway being enriched ($p < 0.05$) in the myeloid dataset. The set of pathways was collated from publicly-available databases including GO and KEGG.

Table S7. Pathways significantly (p<0.05) upregulated in both the combined Track-HD and Leiden whole blood data and the unstimulated myeloid cell dataset of Miller, et al. [1]. Pathways are ordered by their combined p-value, which was obtained by combining the blood and myeloid p-values by Fisher's method.

Table S8. Pathways significantly (p<0.05) downregulated in both the combined Track-HD and Leiden whole blood data and the unstimulated myeloid cell dataset of Miller, et al. [1]. Pathways are ordered by their combined p-value, which was obtained by combining the blood and myeloid p-values by Fisher's method.

Table S9. All WGCNA brain expression modules significantly dysregulated (p < 0.05) in both Track-HD and Leiden datasets in HD versus control blood. *HD brain* modules were defined by Neueder and Bates [2], and *Control brain* modules were derived from Braineac [3] or Gibbs, et al. [4] expression data. Neueder and Bates [2] module identifiers are given in brackets where available. ***** denotes the caudate modules that were highly positively and negatively correlated with HD in their study. *HTT* is part of modules 66 (CNneg1) and 3 (CBneg2). *HD* co-expression modules defined by Neueder and Bates [2]; *CTRL (B)* – control brain co-expression modules from Braineac [3]; *CTRL (G)* – control brain co-expression modules from Gibbs, et al. [4]. *p (Combined/Track-HD/Leiden*) – p value for differential expression between HD and controls in the combined, Track-HD or Leiden datasets; *BH (HD)* the Benjamini Hochberg significance value of correlation with HD in Neueder and Bates [2] brain expression analysis, corrected for multiple comparisons; *Cor (HD)* the direction and size of correlation of a module with HD in Neueder and Bates [2]; *CN* – caudate nucleus; *FC* – frontal cortex;

FC_*BA4* - BA4 region of the frontal cortex; *FC_BA9* – BA9 region of the frontal cortex; *CB* – cerebellum; *TCTX* – temporal cortex.

Table S10. All nominally significantly dysregulated genes (p<0.05) from the WGCNA brain expression modules that were dysregulated (up or down) in HD blood. *p (Comb/Track-HD/Leiden)* – p value for differential expression between HD and controls in the combined, Track-HD or Leiden datasets; *Log2FC* – log2 of the ratio of the mean counts in HD and controls; *HD* co-expression modules defined by Neueder and Bates [2]; *CTRL (B)* – control brain co-expression modules from Braineac [3]; *CTRL (G)* – control brain co-expression modules from Gibbs, et al. [4].

Table S11. Module membership (kME) of genes in module 48 (CNpos2) that are dysregulated in both blood and caudate. There is a significant correlation between the dysregulation of a gene (p value) in the combined Track-HD and Leiden HD blood dataset and its kME, or degree of module membership ($p = 7.6 \times 10^{-4}$). *kME* – correlation of a gene's expression profile with the module eigengene (representative of all gene expression profiles in a module). 0 implies no connection, 1 a strong positive and -1 a strong negative connection to the genes in a module. Highly connected intramodule hub genes have high kME; *Log2FC (blood/caudate)* – log2 of the ratio of the mean counts in HD and controls in our combined blood dataset or the caudate nucleus from Neueder and Bates [2]; *Directional p (blood/caudate)* – directional p value for differential expression between HD and controls in our combined blood dataset or the caudate nucleus from Neueder and Bates [2].

Table S12. Generic pathways significantly upregulated in both HD blood and prefrontal cortex. Comparing gene expression changes in the combined Track-HD and Leiden HD blood dataset with HD prefrontal cortex from Labadorf, et al. [5], a significant ($p < 0.001$) excess of generic pathways are significantly upregulated ($p < 0.05$) in both datasets. *Blood/brain p* the p value for pathway enrichment in HD relative to controls in the combined Track-HD and Leiden blood dataset (*Combined*) or the prefrontal cortex dataset (*Labadorf*).

Table S13. Gene co-expression modules significantly upregulated in both HD blood and prefrontal cortex. Comparison of gene expression changes in the combined Track-HD and Leiden HD blood dataset with HD prefrontal cortex from Labadorf, et al. [5]. *HD brain* modules were defined by Neueder and Bates [2], and *Control brain* modules were generated from Braineac [3] and Gibbs, et al. [4]. Neueder and Bates [2] module identifiers are given in brackets where available. *CN* – caudate nucleus; *FC* – frontal cortex; *FC BA4* – BA4 region of the frontal cortex; *FC BA9* – BA9 region of the frontal cortex; *CB* – cerebellum; *TCTX* – temporal cortex; *Blood/brain p* the p value for module enrichment in HD relative to controls in the combined Track-HD and Leiden blood dataset (*combined*) or the prefrontal cortex dataset (*Labadorf*); *Cor (HD)* the direction and size of correlation of a module with HD in Neueder and Bates [2]; p (HD) – the BH-corrected p value for module enrichment in HD in Neueder and Bates [2].

Table S14. Generic pathways significantly downregulated in both HD blood and prefrontal cortex. Comparing gene expression changes in the combined Track-HD and Leiden HD blood dataset with HD prefrontal cortex from Labadorf, et al. (35), a significant (p = 0.028) excess of generic pathways are significantly downregulated (p < 0.05) in both datasets. *Blood/brain p* – the p value for pathway enrichment in HD relative to controls in the combined Track-HD and Leiden blood dataset (*Combined*) or the prefrontal cortex dataset (*Labadorf*).

Table S15. Gene co-expression modules significantly downregulated in both HD blood and prefrontal cortex. Comparison of gene expression changes in the combined Track-HD and Leiden HD blood dataset with HD prefrontal cortex from Labadorf, et al. [5]. *HD brain* modules were defined by Neueder and Bates [2], and *Control brain* modules were generated from Braineac [3] and Gibbs, et al. [4]. Neueder and Bates [2] module identifiers are given in brackets where available. *CN* – caudate nucleus; *FC* – frontal cortex; *FC BA4* – BA4 region of the frontal cortex; *FC BA9* – BA9 region of the frontal cortex; *CB* – cerebellum; *TCTX* – temporal

cortex; *Blood/brain p* the p value for module enrichment in HD relative to controls in the combined Track-HD and Leiden blood dataset (*combined)* or the prefrontal cortex dataset (*Labadorf*). ; *Cor (HD)* the direction and size of correlation of a module with HD in Neueder and Bates [2]; p (HD) – the p value for module enrichment in HD in Neueder and Bates [2].

Table S16. Correlation between gene expression and TMS in gene positive Track-HD subjects. *p (corr-TMS)* – p value for correlation between expression and TMS; *q (corr-TMS)* – q value shows correction for multiple testing of genes; *Log2(FC)* – the change in log2 (expression) per unit increase of TMS.

Table S17. Enrichment of up or downregulated pathways from HD vs. control blood (Table S2) with TMS in the combined Track-HD and Leiden cohort. *p(combined-diffexp)* – enrichment p-value for upregulated genes in the combined Track-HD and Leiden sample. *p(TRACK-diffexp)* - enrichment p-value for upregulated genes in the Track-HD sample alone. *p(TRACK-TMS)* - enrichment p-value for genes positively correlated with TMS in the TRACK-HD sample.

Table S18. Enrichment of negatively correlated pathways from HD vs. control blood (Table S3) with TMS in the combined Track-HD and Leiden cohort. *p(combined-diffexp)* – enrichment p-value for downregulated genes in the combined Track-HD and Leiden sample. *p(TRACK-diffexp)* - enrichment p-value for downregulated genes in the Track-HD sample alone. *p(TRACK-TMS)* - enrichment p-value for genes negatively correlated with TMS in the TRACK-HD sample.

Table S19. Enrichment of modules from HD vs control blood (Table S9) with TMS in the combined Track-HD and Leiden cohort. Table is sorted by p (TRACK-TMS). *p(combined-diffexp)* – enrichment p-value for downregulated genes in the combined Track-HD and Leiden sample. *p(TRACK-diffexp)* - enrichment p-value for downregulated genes in the Track-HD sample alone. *p(TRACK-TMS)* - enrichment p-value for genes

negatively correlated with TMS in the TRACK-HD sample. *BH (HD)* the Benjamini Hochberg significance value of correlation with HD in Neueder and Bates [2] brain expression analysis, corrected for multiple comparisons; *Cor (HD)* the direction and size of correlation of the module with HD in Neueder and Bates [2]

Table S20. Correlation between genes differentially expressed in HD from Mastrokolias et al [6] and TMS in the Track-HD gene positive subjects. *p(Mastrokolias)* – p-value for correlation between expression and TMS in Mastrokolias et al. *p(TRACK)* – p-value for correlation between expression and TMS in TRACK. *Log2(FC)* – the change in log2 (expression) per unit increase of TMS.

Table S21. WGCNA co-expression modules from the Gibbs, et al. [4] control brain expression dataset significantly associated with late-onset Alzheimer's disease (LOAD) in the IGAP GWAS are upregulated in HD blood. The four immune-related modules that were the most significantly enriched modules in LOAD are also significantly enriched for upregulation in the combined Track-HD and Leiden HD blood dataset. p (IGAP) – p value for enrichment of the gene set between LOAD and controls in the IGAP GWAS; p (Combined/Track-HD/Leiden) – p value for enrichment of the gene set between HD and controls in our HD blood expression dataset.

Table S22. Co-expression modules from Zhang, et al. [7] late-onset Alzheimer's disease (LOAD) brain expression dataset. Several modules, including *yellow* that was the most significantly differentially connected in LOAD, show enrichment for upregulation in the HD blood expression dataset. *Rank (Zhang)* – modules ranked for significance of differential connectivity with LOAD in Zhang, et al. [7]; *p (Combined/Track-HD/Leiden)* – p value for enrichment of the module between HD and controls in our HD blood expression dataset; *q (Comb)* the false discovery rate estimate given by the q-value.

Table S23. Grouping of upregulated pathways from the combined Track-HD and Leiden data for representation in Supplementary Figure S1.

Table S24. Grouping of downregulated pathways from the combined Track-HD and Leiden data for representation in Supplementary Figure S2.

References

1. Miller, J.R. *et al.* RNA-Seq of Huntington's disease patient myeloid cells reveals innate transcriptional dysregulation associated with proinflammatory pathway activation. *Hum Mol Genet* (2016).
2. Neueder, A. & Bates, G.P. A common gene expression signature in Huntington's disease patient brain regions. *BMC Med Genomics* **7**, 60 (2014).
3. Braineac. Braineac - The Brain eQTL Almanac. (2016).
4. Gibbs, J.R. *et al.* Abundant quantitative trait loci exist for DNA methylation and gene expression in human brain. *PLoS Genet* **6**, e1000952 (2010).
5. Labadorf, A. *et al.* RNA Sequence Analysis of Human Huntington Disease Brain Reveals an Extensive Increase in Inflammatory and Developmental Gene Expression. *PLOS ONE* **10**, e0143563 (2015).
6. Mastrokolias, A. *et al.* Huntington's disease biomarker progression profile identified by transcriptome sequencing in peripheral blood. *Eur J Hum Genet* (2015).
7. Zhang, B. *et al.* Integrated systems approach identifies genetic nodes and networks in late-onset Alzheimer's disease. *Cell* **153**, 707-20 (2013).
